# *Galleria mellonella* Larvae as a Model for Investigating Fungal—Host Interactions

**DOI:** 10.3389/ffunb.2022.893494

**Published:** 2022-04-26

**Authors:** Aaron Curtis, Ulrike Binder, Kevin Kavanagh

**Affiliations:** ^1^Department of Biology, Maynooth University, Maynooth, Ireland; ^2^Institute of Hygiene and Medical Microbiology, Medical University Innsbruck, Innsbruck, Austria

**Keywords:** *Galleria mellonella*, *in vivo*, *Candida*, *Aspergillus*, *Cryptococcus*, fungal infection, innate immunity

## Abstract

*Galleria mellonella* larvae have become a widely accepted and utilised infection model due to the functional homology displayed between their immune response to infection and that observed in the mammalian innate immune response. Due to these similarities, comparable results to murine studies can be obtained using *G. mellonella* larvae in assessing the virulence of fungal pathogens and the *in vivo* toxicity or efficacy of anti-fungal agents. This coupled with their low cost, rapid generation of results, and lack of ethical/legal considerations make this model very attractive for analysis of host-pathogen interactions. The larvae of *G. mellonella* have successfully been utilised to analyse various fungal virulence factors including toxin and enzyme production *in vivo* providing in depth analysis of the processes involved in the establishment and progression of fungal pathogens (e.g., *Candida spps, Aspergillus spp., Madurella mycetomatis, Mucormycetes*, and *Cryptococcus neoformans*). A variety of experimental endpoints can be employed including analysis of fungal burdens, alterations in haemocyte density or sub-populations, melanisation, and characterisation of infection progression using proteomic, histological or imaging techniques. Proteomic analysis can provide insights into both sides of the host-pathogen interaction with each respective proteome being analysed independently following infection and extraction of haemolymph from the larvae. *G. mellonella* can also be employed for assessing the efficacy and toxicity of antifungal strategies at concentrations comparable to those used in mammals allowing for early stage investigation of novel compounds and combinations of established therapeutic agents. These numerous applications validate the model for examination of fungal infection and development of therapeutic approaches *in vivo* in compliance with the need to reduce animal models in biological research.

## Introduction

*Galleria mellonella* (the Greater Wax moth) is a member of the order *Lepidoptera* and the family *Pyralidae* (Kwadha et al., 2017). *G. mellonella* is found globally as a pest of bee hives (Jafari et al., 2010). Over the last 20 years *G. mellonella* has become an extremely useful model organism employed for the *in vivo* study of many human fungal pathogens including *Candida albicans, Aspergillus fumigatus*, and *Madurella mycetomatis*. This model has been employed globally and was utilised in over 250 peer-reviewed papers in 2021. The use of insects as models of infection is

in accordance with the 3R policy of replacement, reduction, and refinement of animal utilisation in research (Russell and Burch, 1959).

## Insect Immune Response

**Figure 1:**
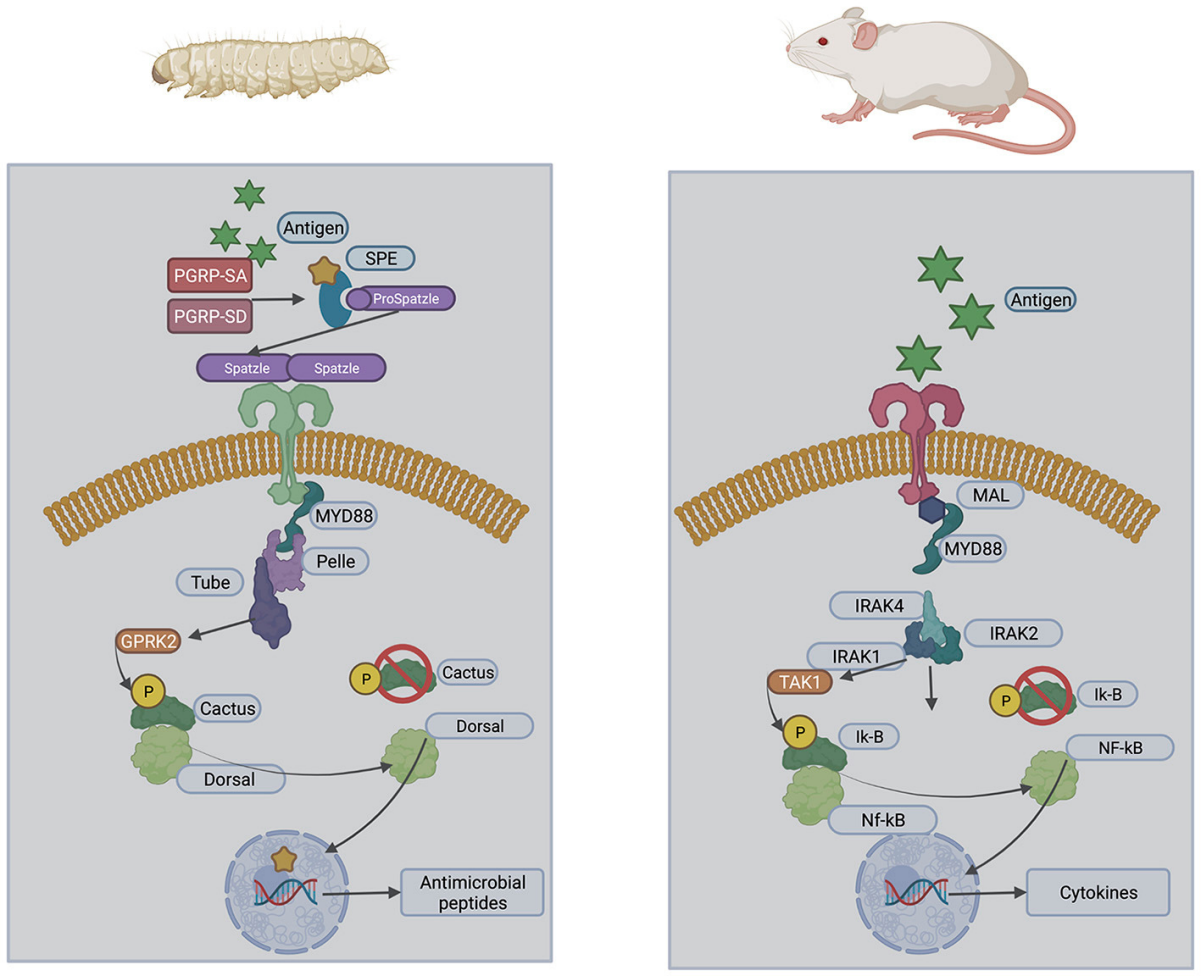
Structural similarities of the insect and mammalian Toll pathways.

The utilisation of *G. mellonella* larvae as a model system is due to the structural and functional similarities between the insect immune response and the innate immune system of mammals (Browne et al., 2013). Insect haemocytes demonstrate specificity and capability to distinguish between classes of microorganisms inducing an appropriate response (Trevijano-Contador and Zaragoza, 2018). This recognition of infection is mediated through germ-line encoded pattern recognition receptors which recognise pathogen-associated molecular patterns (PAMPs) (Lin et al., 2020). These are homologous to those expressed on mammalian innate immune cells (Figure 1) resulting in signalling cascades initiating cellular and humoral immune responses including phagocytosis, nodulation, agglutination, encapsulation, and production of antimicrobial peptides (Lin et al., 2020). Fungal α-1,3-glucan can be recognised by specific recognition proteins which induce antifungal humoral responses when *G. mellonella* larvae were exposed to *Aspergillus niger* resulting in increased expression of antifungal antimicrobial peptides galiomycin and gallerimycin (Staczek et al., 2021). Haemocytes can produce superoxide when activated and have a comparable mechanism to the NADPH oxidase complex of human neutrophils (Bergin et al., 2005). The action of insect haemocytes could also be inhibited in a similar manner to neutrophils following exposure to the mycotoxins gliotoxin and fumagillin produced by *A. fumigatus* (Fallon et al., 2011). This similarity enables comparable results to be obtained to murine models with larval studies yielding data within 2 days compared to 2 months in murine studies (Firacative et al., 2020). The reduced cost of larvae also enables the use of larger test populations resulting in more robust and statistically significant data. *G. mellonella* larvae are unique among insect models as they can be stored at 37°C enabling analysis of human pathogens at biologically relevant temperatures (Fuchs and Mylonakis, 2006).

## Experimental End Points

**Figure 2:**
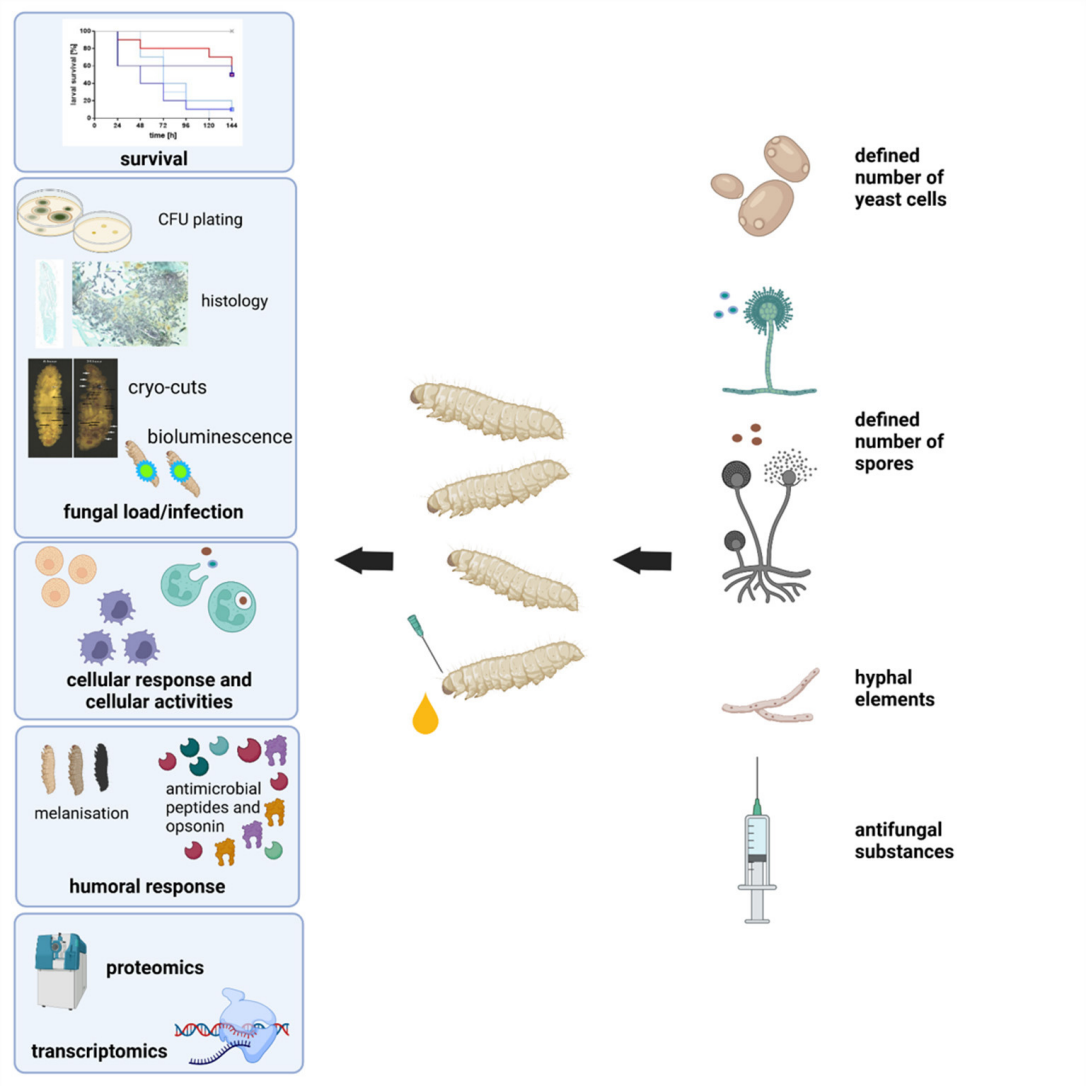
Schematic overview of experimental end-points when using *G. mellonella* larvae as an infection model.

**Figure 3:**
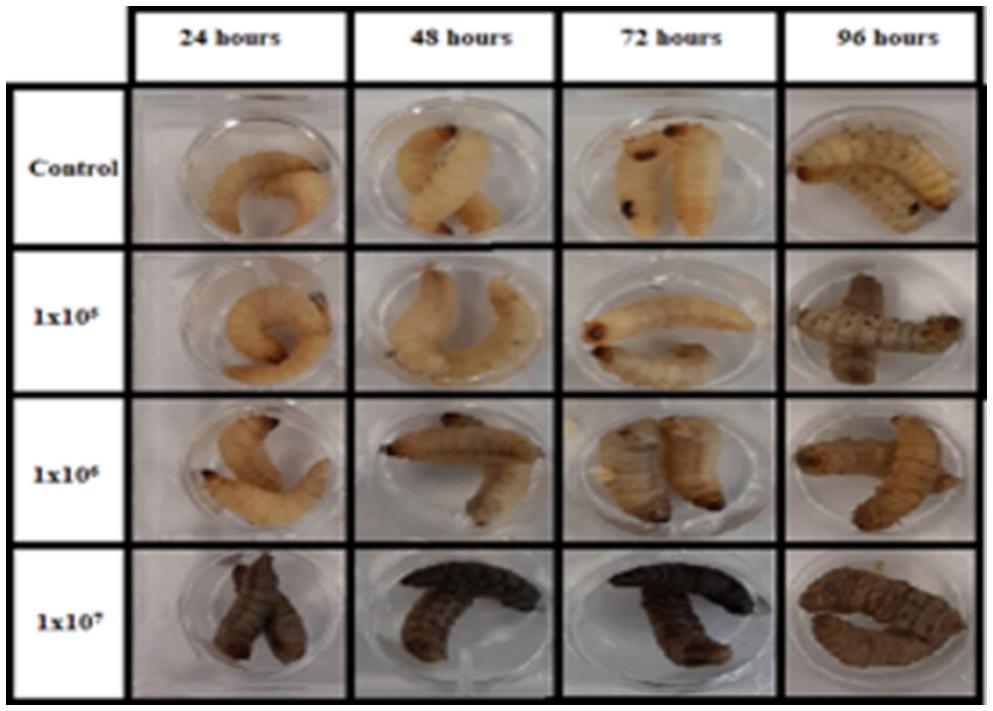
Melanisation of larvae infected with three doses of *A. fumigatus* conidia over 96 h.

**Figure 4:**
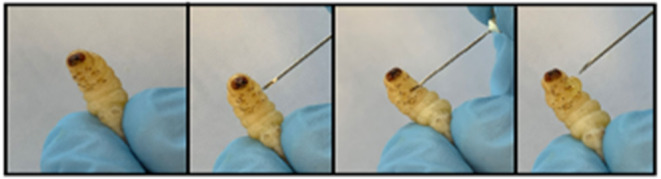
Method for extraction of haemolymph from *Galleria* larva by piercing the second left proleg.

*G. mellonella* larvae may be purchased relatively inexpensively from a variety of commercial suppliers and stored in the laboratory in the dark at 15°C prior to use. It is possible to culture larvae from eggs in the laboratory using diets consisting of oatflakes, dried yeast, glycerol, and honey at 27°C and 60% humidity. Eggs hatch after about 7 days and sixth instar larvae are obtained ~6 weeks after hatching. There is a variety of endpoints to experimentation in larvae which facilitate the rapid assessment of infection or the efficacy of a therapeutic strategy (Figure 2). Melanin is a toxic compound thus its production is tightly regulated (Nakhleh et al., 2017) and melanisation occurs following activation of the prophenoloxidase cascade triggered following microbial entry which activates serine proteases (Pereira et al., 2018) (Figure 3). Movement can also serve as an indicator of larval viability as the ability to self-right or turn around are indicators of health (Durieux et al., 2021). Larval movement can be utilised to track changes in behaviour following exposure to toxins and compounds as demonstrated following administration of caffeine which resulted in inhibition of movement due to the presence of the metabolite theophylline (Maguire et al., 2017). Cocoon formation is an indicator of larval fitness and life progression, *G. mellonella* larvae infected with *Candida auris* showed reduced cocoon formation which correlated with the virulence of the strain (Romera et al., 2020). In addition, alterations in haemocyte density can serve as an endpoint for determining larval response to pathogens and chemicals (Figure 4). *G. mellonella* larvae inoculated with various strains of *C. neoformans* demonstrated a 7-fold increase in haemocyte density 2 h post infection, however this was not observed when strains devoid of the capsule were inoculated (García-Rodas et al., 2011). The quantification of haemocyte density can be used to determine the response to chemicals and antimicrobials and can serve as an initial screening system. Novel Cu(II) phenanthroline-phenazine complexes at an LD_50_ concentration did not alter the haemocyte density indicating the clearance of infection was due to the action of the compound alone (Rochford et al., 2018). Analysis of changes in fungal burden can also be conducted to determine the response of the larvae to a pathogen. This involves the introduction of a known quantity of fungal cells into larvae followed by the homogenization of larvae and serial dilution of samples onto agar plates to determine the level of killing. *G. mellonella* infected with *C. auris* displayed a higher fungal burden compared to those infected with other *Candida* strains and the results suggested that *C. auris* strains were more virulent in larvae when compared to other members of the *C. haemulonii* complex (Muñoz et al., 2020). Fungal toxin production is another experimental endpoint and can be quantified *in vivo*. A clinical *A. fumigatus* strain produced higher larval mortality at 48 h and produced the highest concentration of gliotoxin indicating a positive correlation between gliotoxin production and virulence (Reeves et al., 2004). The action of fumagillin on insect haemocytes was characterised and treated cells demonstrated reduced degranulation, phagocytic activity, and ability to produce reactive oxygen species indicating a similar response to neutrophils (Fallon et al., 2010). Variations in gene expression particularly in genes of the larval immune response can also be utilised to access the state of infection of individuals. These genes can consist of antimicrobial peptide encoding genes at various tissue locations including the fat body and midgut, and the expression can be quantified using qPCR methods (Kryukov et al., 2020). The rate of gene expression can also be influenced by environmental factors particularly temperature (Mowlds and Kavanagh, 2007).

## Visualisation of Fungal Infections

**Figure 5:**
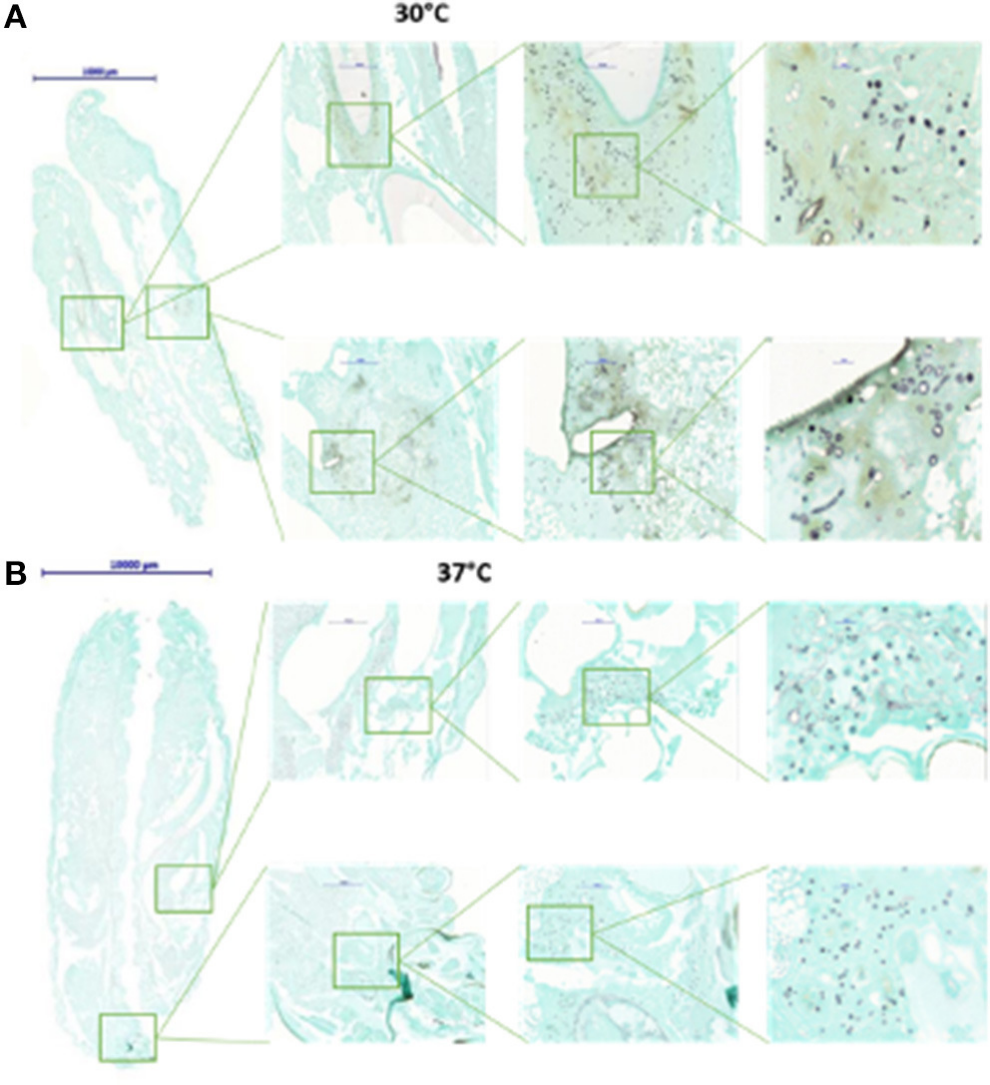
Histological examination of *G. mellonella* larvae infected with *Mucor circinelloides*. Larvae were fixed in formalin 72 h after infection, embedded in paraffin. Tissue sections were prepared at a thickness of 3.0 μm and stained with Grocott silver stain to optimise visualisation of fungal elements. When larvae are incubated at 30°C **(A)**, germlings and hyphal elements are detected in higher abundance than when incubated at 37°C **(B)**.

One important end point in many investigations using *G. mellonella* larvae, or animal models in general, is the determination of fungal burden at certain time points after infection or after the application of an antifungal drug. For yeasts and bacteria microbial burden is often determined by homogenization of infected larvae and subsequent plating of the diluted material onto agar plates to calculate viable cells via the formation of CFUs. This method works very well for unicellular organisms but in the case of filamentous moulds has certain drawbacks. Another option would be quantifying the amount of fungus via qPCR, but this is expensive and time consuming. Another option to get an idea of the fungal burden in infected hosts is tissue analysis. It will not give numbers to compare, but certainly has the advantage of also giving insights into the *G. mellonella* larval response to the infection. Cryo-imaging of larvae infected with both *C. albicans* and *S. aureus* revealed the formation of melanized nodules, first at the site of infection, but then disseminated throughout the larvae which was in agreement with upregulation of genes involved in nodule formation (Sheehan et al., 2020). Microsections of formaldehyde fixed and paraffin embedded larvae that were stained with Grocott's silver stain, showed correlation between increased hyphal mass simultaneously to increased deaths and melanisation in larvae infected with *A. terreus* (Maurer et al., 2015). Similar data were obtained for *G. mellonella* larvae infected with different species of Mucormycetes (Maurer et al., 2018) (Figure 5). Applying any of these methods means that specimens have to be sacrificed at every time point. One method that would prevent this, is the usage of bioluminescent reporter strains and attempts to prove their suitability have already been made in *G. mellonella* larvae. Delarze et al. adapted the Gaussia princeps luciferase reporter system for *in vivo* detection of *C. albicans* infections in *Galleria* larvae. They followed two principles, either measuring and imaging light emission in homogenised galleria/larval pulp, resembling an *ex vivo* system or imaging light emission in viable *G. mellonella* larvae with CCD cameras. In this system light intensity correlates with fungal metabolism and biomass. *G. mellonella* larvae that had been infected with several transcription factor mutants produced reduced light intensity when infected with strains exhibiting lower virulence potential and lower CFU counts (Delarze et al., 2015). Additionally, the efficacy of fluconazole was compared by survival analysis, CFU counting and detecting luminescence and a positive correlation was seen. Luminescence signals were 10x lower in larvae receiving antifungal treatment compared to the untreated control larvae (Delarze et al., 2015). For mould pathogens similar attempts were carried out with *Mucor circinelloides*, which was successfully modified to express firefly luciferase. Although the strains could nicely be utilised to determine *in vitro* antifungal susceptibility by other means than MIC reading, the mammalian optimised sequence was not optimal for use in *G. mellonella* larvae. In a follow up project *Mucor* strains expressing codon optimised luciferase were generated and were suitable for *in vivo* light detection in infected larvae (Cornely et al., 2021). The technique of bioluminescent imaging provides a very useful tool to follow initiation and progression of infection in individual hosts as well as visualise the efficacy of antifungal drugs.

## Mechanism Employed by Fungi to Infect *Galleria* Larvae

All species experience co-evolution with pathogenic species with defence mechanisms and virulence factors evolving to protect and infect a host, respectively (Joop and Vilcinskas, 2016). Similar to other insects, *G. mellonella* has developed natural defences against environmental fungal pathogens such as Beauveria bassiana, which is commonly utilised as a biocontrol agent (Lacey et al., 2015). Entry via the cuticle is facilitated through the secretion of cuticle secreting enzymes (Dhawan and Joshi, 2017). When growing in haemolymph B. bassiana adopts a yeast-like structure with thin blastospores which aid in immune evasion within the host (Tartar et al., 2005). In response to B. bassiana invasion larvae produce melanin and there is a reduction in the plasmatocyte population possibly indicating an inhibition of differentiation induced by the fungus (Vertyporokh et al., 2019). Secondary metabolites secreted by *B. bassiana* such as oosporein have been demonstrated to be immunosuppressive thus facilitating fungal infection (Mc Namara et al., 2019).

*G. mellonella* larvae can also be utilised to evaluate infections by many clinically relevant species. The introduction of the pathogen is typically facilitated via intra-haemocoel injection of larvae weighing between 250 and 300 mg that show no signs of melanisation and display activity (Pereira and Rossi, 2020). The dose can vary in volume between 10 and 40 μL and typically utilises a Hamilton or fine tip insulin syringe to deliver an injection through the last left proleg of the larva (Champion et al., 2018). Third instar larvae that are actively feeding can be fed microbes by drenching feed with microbial cultures to mimic a physiological route of natural exposure to pathogens (Freitak et al., 2014). Oral gavage can also be utilised for introduction of pathogen or compound of interest using a blunted microinjector syringe inserted into the mouthparts of final instar larvae to deliver a 20 μL inoculum (Maguire et al., 2017).

## Development of Yeast in *Galleria* Larvae

The virulence factors contributing to colonisation by pathogenic yeast including those belonging to the *Candida* and *Cryptococcus* genus have been examined in *G. mellonella*. The crepe and rough phenotypes of *C. tropicalis* induced higher levels of melanisation in larvae 24 h post infection suggesting phenotypic switching of yeast may produce structures that are detected differently in *G. mellonella* larvae. The crepe phenotype was observed to be phagocytosed more readily than the parental strain while the rough phenotype was less readily phagocytosed (Perini et al., 2019). The role of filamentation of *C. albicans* was also assessed in *G. mellonella* larvae through examination of five mutant strains deficient in genes (i.e., *bcr1, flo8, kem1, suv3, and tec1*) that have been shown to play a role in filamentation and biofilm formation. FLO8 mutants were not capable of forming filaments *in vivo* and demonstrated reduced virulence in larvae. Nevertheless, inhibition of filamentation alone was not sufficient to prevent virulence indicating other factors are associated with mortality (Fuchs et al., 2010). There was a statistically significant correlation between the level of phospholipase activity *in vitro* and the killing of larvae i*n vivo* indicating the ability to produce these enzymes increases the virulence of the species (Rossoni et al., 2013). These virulence factors facilitate the adherence, invasion and development of *Candida in vivo*.

Proteomic analysis can be conducted on haemolymph of infected *G. mellonella* larvae to examine the secreted and structural fungal proteins to provide insight into the early adaptations to facilitate infection. Proteins that defend against aspects of the immune response were increased in abundance and included heat shock protein SSA1 which binds to antimicrobial peptides and antigenic secreted protein RBT4 which acts as a virulence factor during infections and plays a role in protection against phagocyte attack (Sheehan and Kavanagh, 2019). Analysis of the response of *C. albicans* incubated *ex vivo* in *G. mellonella* haemolymph for 6 h demonstrated alterations in the proteomic response and a significant increase in abundance of proteins associated with the stress response, glycolysis and the citric acid cycle, while abundance of protein associated with translation, ribosomal activity was significantly decreased. Proteins associated with an oxidative stress response such as thioredoxin, superoxide dismutase, glutathione-disulfide reductase were increased in abundance indicating cellular stress was induced in the haemolymph (Sheehan and Kavanagh, 2019). This analysis indicates a similar response of *C. albicans* in haemolymph and the bloodstream of mice and indicate which processes are involved in facilitating the infection.

The *C. neoformans* polysaccharide capsule, as well as several *C. neoformans* genes previously shown to be involved in mammalian virulence including gpa*1, pka1*, and *ras1*, were shown to play a role in the killing of *G. mellonella* larvae (Mylonakis et al., 2005). The presence of the capsule was also influenced the lytic response in larval haemolymph (Trevijano-Contador et al., 2014).

## Development of Filamentous Fungi in *Galleria* Larvae

*A. fumigatus* deletion mutants of genes involved in siderophore biosynthesis (*sidA, sidF*), or encoding para-aminobenzoic acid synthetase *(paba)*, catalysing a late step in folate biosynthesis, were avirulent in *G. mellonella* larvae while deletion mutants of non-ribosomal peptide synthetases, *sidC*, and *sidD*, demonstrated reduced virulence and the results were comparable with data from murine studies (Slater et al., 2011). Adherence of *A. fumigatus* to host tissue is driven by the conserved -terminal domain MedA which regulates conidiogenesis, adherence to host cells, and pathogenicity. The mutants lacking this region demonstrated impaired biofilm formation and reduced adherence capacity in pulmonary epithelial cells *in vitro* and reduced virulence in murine models of invasive aspergillosis and in *G. mellonella* larvae (Al Abdallah et al., 2012). Pigmentation of conidia, through production of melanin provides protection against reactive oxygen species produced by the host and the six gene cluster *alb1, arp2, arp1, abr1, abr2, and ayg1* is required for *A. fumigatus* pigmentation (Al Abdallah et al., 2012). *Alb1, abr2*, and *arp1* mutants were highly virulent in *G. mellonella* larvae and other colour mutants such as *arp2* and *abr1* were at least as virulent as the wild type strain. The mutants displayed varied sensitivity toward hydrogen peroxide and the *alb1* mutant in B5233 background was most sensitive to hydrogen peroxide followed by the *ayg1* mutant (Al Abdallah et al., 2012). Although sensitivity toward hydrogen peroxide does not appear to be a good indication of pathogenicity in *G. mellonella*, it is likely that this resistance further increases *A. fumigatus* virulence potential. In mice, colour mutants *alb1* and *arp1* induce a more robust immune response, resulting in reduced virulence (Jackson et al., 2009). It has been speculated that the exaggerated immune response of *G. mellonella* larvae to *A. fumigatus* colour mutants is one of the major factors responsible for the morbidity of these larvae, possibly triggered by the altered surface properties of the colour mutant conidia, inducing an over-reactive immune response preventing clearing of conidia (Jackson et al., 2009). In addition, the role of cofilin overexpression in *A. fumigatus* was assessed using mice and *G. mellonella* larvae and showed that, due to the protein's role in oxidative stress resistance, the overexpression mutant was also internalised more efficiently by lung epithelial cells and increased polysaccharide production resulting in increased PAMP activation and immune activation. Despite this variation, overexpression of cofilin did not increase the virulence in either murine or larval infection (Jia et al., 2017). Secreted aspartic proteases have been implicated as a virulence factor facilitating tissue invasion by fungal pathogens. CtsD, an aspartic protease distinct to *A. fumigatus* was examined as a virulence factor in *G. mellonella* larvae (Vickers et al., 2007). Larvae infected with conidia had a higher mortality rate than larvae infected following treatment with an anti-CtsD antibody indicating the enzyme is produced and secreted during infection and has a potential role in facilitating virulence of the fungus *in vivo* (Vickers et al., 2007). Both fumagillin and gliotoxin affect the immune response of *G. mellonella* larvae and have been detected in larvae post infection. Fumagillin induces similar effects in insect haemocytes thus demonstrating further similarities between the two cell types (Fallon et al., 2011). Gliotoxin can be quantified using HPLC *in vivo* and *ex vivo* in *Galleria* larvae and the level of gliotoxin secretion was found to correlate with virulence in larvae whereas elastase, catalase and growth rate did not (Reeves et al., 2004).

During the mammalian innate response to *A. fumigatus*, a range of antimicrobial peptides such as defensins and cathelicidins and proteins including lactoferrin, lysozyme are produced and these are essential in preventing early fungal establishment and growth. At 24 h post infection of *G. mellonella* larvae antimicrobial peptides and prophenoloxidase family proteins were increased in abundance in addition to proteins associated with pathogen recognition and opsonization and inhibition of fungal proteinases, insect metalloproteinase inhibitor (Sheehan et al., 2018).

**Figure 6:**
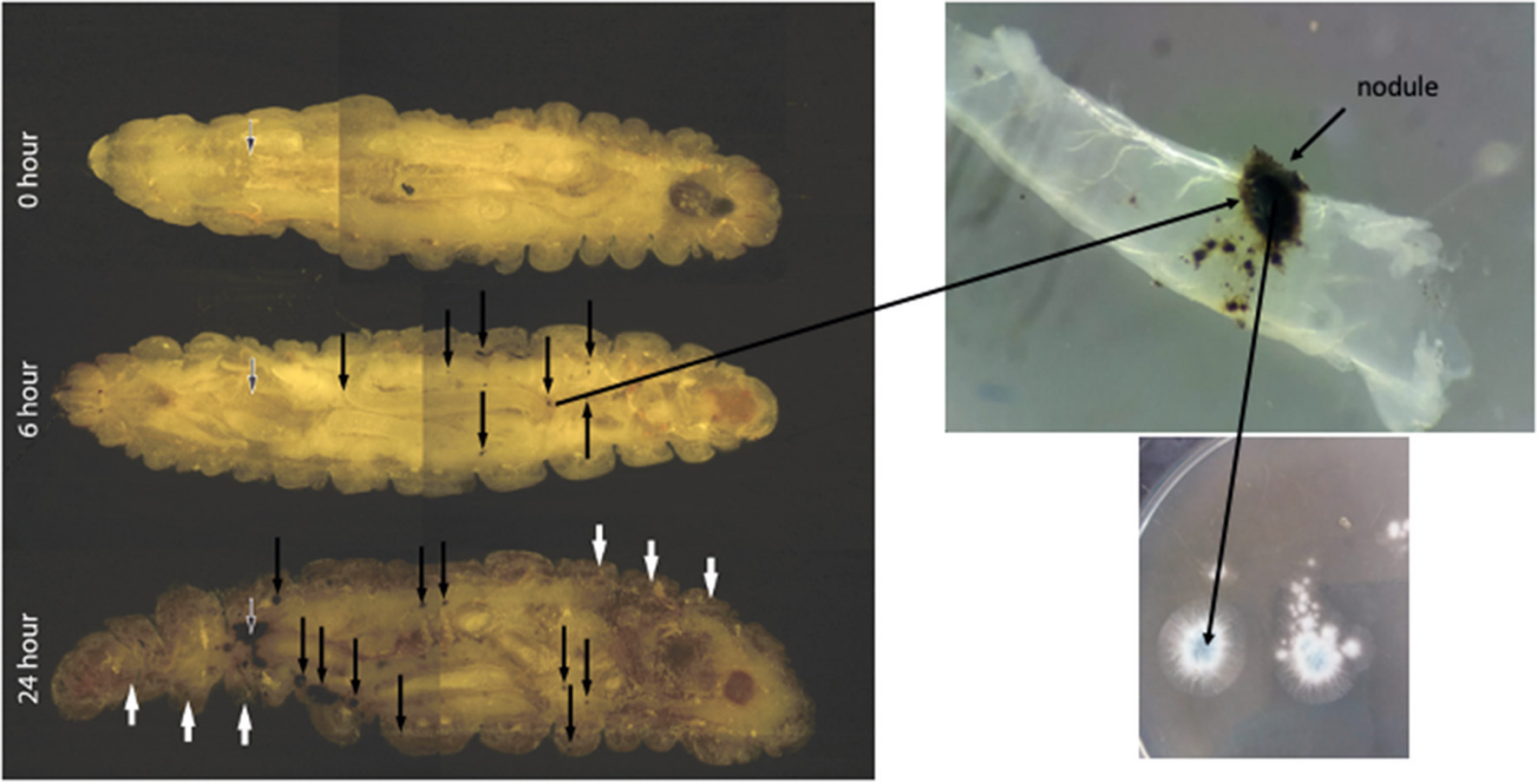
Cryoviz visualisation of the stages of invasive and disseminated aspergillosis in *G. mellonella* larvae after 6 and 24 h infection. Larvae were inoculated with 1 × 10^6^ *A. fumigatus* conidia and embedded in Cryo-imaging embedding compound and sectioned (10 μm) using a Cryoviz™ cryo-imaging system (Fungal nodules—black arrows; cuticle melanisation- white arrows; point of inoculation—white edged black arrow) (Image courtesy of Dr. Gerard Sheehan).

The stages of *A. fumigatus* invasion *in vivo* can be tracked as the disease progresses utilising cryo-imaging. Small discrete nodules appeared in the anterior region and around the perimeter of the haemocoel 6 h post infection indicating dissemination of the *A. fumigatus* conidia from the site of infection (Sheehan et al., 2018). Following 24 h of infection extensive melanization of larval tissue and cuticle was present indicating invasion from the insect haemocoel into surrounding tissue. Confocal laser scanning microscopy of nodules isolated at 6 and 24 h confirmed the presence of germinated conidia at 6 h and dense hyphal infiltration at 24 h post infection (Sheehan et al., 2018) (Figure 6).

The tropical fungus *Madurella mycetomatis* is the causative agent of eumycetoma and grain development can be tracked using histology on fixed and dissected larvae (Sheehan et al., 2019). Initially the larval response to pathogen entry involved activation of the nodulation response resulting in increased abundance of proteins associated with tissue disruption due to fungal proliferation and hyphal formation (Sheehan et al., 2019). Initial *M. mycetomatis* protein alterations were associated with grain formation and proteins associated with vesicle transport, including proteins associated with the secretory vesicles in the endoplasmic reticulum and the Golgi apparatus which were present in both haemolymph and fungal grains (Sheehan et al., 2019). After recognising the pathogen, *G. mellonella* haemocytes begin to agglutinate around the pathogen forming an overlapping sheath. One of the proteins which plays an essential role in the crosslinking of haemocytes and pathogens during nodule formation is Noduler, or the *G. mellonella* homologue Hdd11. *M. mycetomatis* itself also seems to play a role in the cross-linking of the extracellular matrix (Sheehan et al., 2019). In the day 1 grain proteome the Asp2f homologue was found. This protein is secreted from the fungal cell to form a complex with extracellular zinc and is recruited back to the fungal cell. Elevated levels of zinc have been noted within mycetoma grains in humans, which could indicate that a similar cross-linking activity of Asp2f also takes place in the production of the cement material noted in the *M. mycetomatis* grains. Aggregation of granular cells followed by degranulation is typical for the *G. mellonella* nodule formation and leads to the accumulation of coagulogen around the fungus followed by melanisation (Sheehan et al., 2019). Antimicrobial peptides were increased in 24 h grains relative to control haemolymph, while cationic peptide CP8 was decreased in abundance at this time point. Lysozyme was found highly enriched within *M. mycetomatis* grains but absent in haemolymph which may confirm that certain proteins are shuttled from the haemolymph to the site of infection (Sheehan et al., 2019). During degranulation of the haemocytes prophenoloxidase is released and this initiates the melanisation process of the *G. mellonella* nodule. In the *M. mycetomatis* grains found in human, DHN-melanin is present and the cement material itself is melanised. It is highly likely that in *G. mellonella*, the grain is melanised via both the *G. mellonella* proPO pathway and the *M. mycetomatis* DHN-melanin pathway (Sheehan et al., 2019). After melanisation, the encapsulation process is often terminated by forming a basement membrane like layer around the capsule periphery (Sheehan et al., 2019).

Infection with conidia of *Talaromyces marneffei* resulted in a dose dependent killing of larvae and it was demonstrated that FITC labelled conidia were gathered inside haemocytes 2 h post infection indicating the role of phagocytes in the clearing of infection (Huang et al., 2015). The temperature dependent dimorphic switch from mycelial growth to fission yeast morphology was examined in larvae with *rttA* mutants displaying inappropriate transition resulting in reduced virulence (Suwunnakorn et al., 2014). The role of another form of dimorphism, namely size dimorphism of *Mucor circinelloides* spores, was studied by Li et al. (2011) who could correlate greater size and irregular shaping of spores produced by the (-) mating type isolates to higher virulence potential in *G. mellonella* larvae. It was hypothesised that a larger fungal biomass challenges the larval immune system or a difference in immune response to the different morphologies may occur (Li et al., 2011). Assays using murine macrophages showed that big spores of (-) mating type isolates germinated inside the macrophages and also germinated and grew much faster in presence of macrophages than their smaller counterparts (Li et al., 2011). Although the authors did not investigate the fate of spores in hemocytes, a similar mechanism could occur and lead to faster killing of infected larvae (Li et al., 2011).

*Aspergillus terreus* infections have been intensively studied in the *G. mellonella* larvae. Comparison of survival of larvae infected with 73 different *A. terreus* isolates of “so called” cryptic species revealed similar virulence potential as the *A. terreus sensu stricto* isolates (Lackner et al., 2019). Differences in virulence were shown to be dose and temperature dependent, and, for the few survival rates that differed from the average survival rates, found to be rather strain than species dependent (Lackner et al., 2019). Furthermore, no correlation was found between survival rates of infected larvae and the source of the isolate, e.g., human patient isolate or environmental (Lackner et al., 2019). Nevertheless, survival rates of specific clinical *A. terreus* isolates that exhibited amphotericin B susceptibility could be linked to significantly faster germination and growth by these susceptible isolates, plus weaker immune defences against these isolates. This was evident by reduced damage of hyphae caused by isolated hemocytes against these strains, plus lower numbers of circulating hemocytes 48 h post infection (Maurer et al., 2015).

## Elucidating Co-infection by the Use of *G. mellonella* Larvae

In the clinical setting polymicrobial infections or a dysbalance of the human microbiome are often the most challenging cases to treat. Therefore, it is essential to understand host response during co-infections in detail. The airways of cystic fibrosis (CF) patients are considered to be the habitat of many different microbes that play an important role in disease pathology. Two such microbes prominently found in CF lungs are *Pseudomonas aeruginosa* and *A. fumigatus*. Priming the immune system of larvae by sub-lethal concentrations of *A. fumigatus* (100 conidia), to mimic colonisation, resulted in significantly higher mortality rates in groups subsequently injected with 2 × 10^5^ *P. aeruginosa* cells 24 h after inoculation with *A. fumigatus* (Reece et al., 2018). The presence of *P. aeruginosa* supports *Aspergillus* growth by the production of volatile sulphur compounds that provide a sulphur source to the fungus (Scott et al., 2019). In the case of *C. albicans* and *S. aureus* co-infection, co-infection caused disseminated disease throughout the larvae and the establishment of large nodules, which was different to infection with only one of the pathogens. Increased mortality was related to increased amount of proteins involved in immune response in *G. mellonella* hemolymph, such as cecropin-A (Sheehan et al., 2020).

## Transcriptional Analysis of Pathogens and the Larval Host

In the last decade, since the generation of the first comprehensive transcriptome of *G. mellonella* by Vogel and colleagues, substantial information on the biology of and response to pathogens was acquired by genomic and transcriptomic studies. A similarity of ~40% to proteins of other insects was identified and the function of about half was described (Vogel et al., 2011). With the first genome sequence obtained in 2018, hopes were high for broadening the use of *G. mellonella* larvae (Lange et al., 2018). Unfortunately, detailed functional annotation and analysis is still missing which limits comparison to other, well-established model systems and would facilitate getting a full picture of host-pathogen interactions (Lange et al., 2018). The analysis of the *C. albicans* transcriptome during infection of *G. mellonella* larvae and mice revealed that many of the genes are expressed similarly in both model hosts and the transcriptional patterns did not differ greatly between early (2 and 16 h post infection) and late-stage (24 and 48 h) infection (Amorim-Vaz et al., 2015). Among the 20 most upregulated genes in both models were genes involved in cell host adhesion, invasion, dissemination, hyphal development, and iron metabolism. Additionally, to these pathways, that were expected to be upregulated, a large group of genes was found to be differential expressed whose functions are still uncharacterised (Amorim-Vaz et al., 2015). Most importantly, the similarity in expression patterns between the invertebrate and the murine model underlines the importance and relevance of the larval model and facilitates its usage in place of vertebrate models.

Next to human pathogenic fungi, expression analysis of the natural insect pathogen *B. bassiana* was investigated. Transcriptome analysis was performed on *G. mellonella* larvae at three time points post infection and revealed thousands of differentially expressed genes at each time point (Chen et al., 2018). Additionally, to RNASeq gene expression of selected genes was quantified with qRT-PCR which led to a categorisation into four different patterns from genes highly expressed across the entire infection, highly expressed during early infection, but low at the end, or the opposite and genes that showed low expression rated throughout infection process (Chen et al., 2018). Data obtained that help understanding pathogenic mechanisms, especially if they are comparable in the individual host system, are essential to design strategies for future drug development. Furthermore, with the increasing ethical constrains of using vertebrates, characterising the suitability of the *G. mellonella* model is crucial.

## Use of *G. mellonella* Larvae for Evaluation of Antifungal Strategies

*G. mellonella* larvae can be utilised to examine the *in vivo* efficacy of antifungal therapeutics following experimental induction of infection in larvae. Larvae infected with *C. tropicalis* and treated with fluconazole, amphotericin B and caspofungin were protected from infection (Silva et al., 2018). Following *C. krusei* infection, fluconazole did not protect the larvae at a high or low concentration while amphotericin B and caspofungin had a protective effect. Larvae infected with *C. lusitaniae* were protected from infection by both fluconazole and caspofungin at all tested concentrations, whereas amphotericin B only provided protection at the highest concentration tested (Silva et al., 2018). In the case of *C. haemulonii* species complex infections, only caspofungin had a protective effect. None of the drugs was toxic to the *G. mellonella* larvae at the tested concentrations (Silva et al., 2018). Positive correlations between *in vitro* susceptibility data and *in vivo* efficacy of fluconazole against *C. parapsilosis* and *C. orthopsilosis* have been demonstrated (Morio et al., 2019; Binder et al., 2020). While survival of larvae infected with fluconazole susceptible wildtype strains could be significantly prolonged by a single dose of fluconazole, no improvement in survival was observed in larvae infected with azole resistant clinical *C. orthopsilosis* isolates that harboured a G458S amino acid exchange in the erg11 gene. Similarly, *C. parapsilosis* isolates exhibiting multidrug (azole and echinocandin) or azole resistance *in vitro*, did not respond to fluconazole or micafungin treatment *in vivo*. Nevertheless, positive treatment outcome was observed in all cases when the treatment regime was a single dose of liposomal amphotericin B. Drug dosing was chosen according to the dosage recommended by the European centre of antimicrobial susceptibility testing (EUCAST rational version 3.0) and these concentrations were well-tolerated by the larvae (Binder et al., 2020).

The pharmacokinetics of antifungals can also be examined *in vivo* with studies describing a linear increase in C_mas_ and AUC_0−24_ in larval haemolymph which is similar to human serum, the distribution was also found to be similar with the derived volume of distribution being almost identical between humans and larvae (Astvad et al., 2017). Some differences emerged regarding metabolism and half-life as fluconazole is primarily excreted by the kidneys, which are absent in insect. However, the half-life in larvae was about a third of the human value showing greater clearance over time possibly due to the action of the larval fat body (Astvad et al., 2017). Larvae infected with *C. auris*, an emerging yeast pathogen that is often associated with antifungal resistance, could successfully be rescued by NSC319726. This drug is a thiosemicarbazone zinc chelator that, besides inhibiting growth of mammalian cancer cells with p53 mutation, was shown to have growth inhibitory effect also against various fungal pathogens (Sun et al., 2017; Li et al., 2021).

Efficacy of antifungal compounds on filamentous fungi was also evaluated in larvae. Amphotericin B has been utilised for treatment of invasive aspergillosis in *G. mellonella* and larvae treated with amphotericin B at 3 mg/kg had a 90% survival rate. Amphotericin B, or its liposomal version, also showed efficacy against rare susceptible *A. terreus* isolates in *in vivo* studies, again showing good correlation of *in vitro* and *in vivo* (*G. mellonella* and murine) data when using clinical relevant dosage (Maurer et al., 2015). Moreover, a recent study indicated that the occurrence of drug tolerance in amphotericin B susceptible isolates could explain poor clinical outcome, as although strains that exhibited MIC values that would classify them as susceptible, were able to propagate under amphotericin B pressure, and infected larvae could not be cured by drug treatment (Vahedi-Shahandashti et al., 2022). Voriconazole was also effective in treating aspergillosis and was observed as being more effective in larvae infected with susceptible isolates compared to those infected with a resistant isolate (Jemel et al., 2021).

The novel nystatin formulation, nystatin-intralipid, exhibited best activity against Mucorales, followed by posaconazole, while limited efficacy was seen for liposomal amphotericin B and isavuconazole in larvae. Furthermore, nystatin-intralipid did not affect hemocyte density; therefore, it can be assumed that the increase in survival was mediated by inhibiting fungal growth through exerting its antifungal activity and not through an unspecific immune response (Maurer et al., 2018). The *in vivo* efficacy of combinational therapy has been examined validating the synergy between amphotericin B and flucytosine against *C. albicans* (Li et al., 2013). While monotherapy by amphotericin B or flucytosine was not effective, the combination of the two drugs significantly improved survival of infected larvae. Combination of amphotericin B with flucytosine is the recognised therapy for cryptococcosis and also used for difficult-to-treat invasive candidiasis such as endocarditis, endophthalmitis, and meningitis which could be examined utilising the *G. mellonella* model (Jemel et al., 2020).

Combination of amphotericin B with a HSP70 inhibitor pifithrin-μ had a synergistic effect with increasing survival rates in treated *A. terreus* infected larvae compared to those injected with only one drug (Blatzer et al., 2015). *G. mellonella* larvae were also employed to study the role of the so-called “MIC phenomena” in *in vivo* efficacy. *C. tropicalis* isolates, that are known to exhibit trailing phenomena (defined as growth inhibition of only 50–80% at high antifungal concentrations) in standardised susceptibility tests, were found to respond less to fluconazole treatment. The higher the degree of trailing, the more fluconazole efficacy was reduced *in vivo*. Similar results were obtained with murine models, although in mice only the strains classified as showing “heavily trailing” were resistant to fluconazole treatment, which might be attributed to the single dose regime in larvae vs. the multiple dosing in mice (Sanglard et al., 2018). That trailing does play a role in antifungal efficacy was undermined by a study showing failure of voriconazole treatment of *C. albicans* infected larvae that demonstrated trailing against the drug *in vitro* (Binder et al., 2019).

## Conclusion

Larvae of *G. mellonella* are now widely accepted and used in academia and industry as a model system for assessing the virulence of microbial pathogens and for determining the *in vivo* toxicity and efficacy of antimicrobial drugs. Larvae offer many advantages over the use of conventional vertebrates screening systems but in certain cases results obtained in *G. mellonella* larvae may require confirmation in a vertebrate system. The advantages of ease of inoculation, rapid generation of results, inexpensive purchasing and housing, and absence of legal/ethical restrictions mean that *G. mellonella* larvae are a popular choice with a wide range of applications. *G. mellonealla* larvae are susceptible to infection by a wide variety of clinically relevant fungal pathogens and show pathologies that display strong similarities to those found in infected mammals. The ability to arrest pathogen development *in vivo* by administering antifungal agents offers the possibility of assessing *in vivo* efficacy and toxicity of novel compounds prior to mammalian testing. Like any model system, the use of *G. mellonella* larvae does have disadvantages or limitations. For example larvae lack an adaptive immune system and many of the organs affected in cases of systemic fungal infection (e.g., lung, kidney, spleen). In addition, results obtained using *G. mellonella* larvae often need confirmation in other systems. However, while recognising these limitations, it is widely accepted that *G. mellonella* larvae are a convenient and cost effective screening system with a wide range of applications. The recent application of proteomic, transcriptomic and imaging techniques to *G. mellonella* larvae increases the attractiveness of the model by creating a wide range of assay end-points as well as fully characterising the host-pathogen interactome. While no model system is perfect, the use of *G. mellonella* larvae offers many advantages and the continual development of this system is warranted.

## Author Contributions

AC and UB wrote the manuscript. KK edited and proof read. All authors contributed to the article and approved the submitted version.

## Funding

AC was funded by an Irish Research Council Government of Ireland Ph.D. scholarship.

## Conflict of Interest

The authors declare that the research was conducted in the absence of any commercial or financial relationships that could be construed as a potential conflict of interest.

## Publisher's Note

All claims expressed in this article are solely those of the authors and do not necessarily represent those of their affiliated organizations, or those of the publisher, the editors and the reviewers. Any product that may be evaluated in this article, or claim that may be made by its manufacturer, is not guaranteed or endorsed by the publisher.
